# AE solutions to two-sided interval linear systems over max-plus algebra

**DOI:** 10.1186/s13660-018-1869-6

**Published:** 2018-10-25

**Authors:** Lihua Wang, Wei Li, Haohao Li

**Affiliations:** 1 Institute of Operational Research & Cybernetics, Hangzhou Dianzi University, Hangzhou, China; 2 School of Data Sciences, Zhejiang University of Finance and Economics, Hangzhou, China

**Keywords:** Two-sided interval linear systems, Max-plus algebra, AE solutions

## Abstract

This paper introduces a concept of AE solutions to two-sided interval max-plus linear systems, a rather general concept which includes many known concepts of solutions to interval systems, in particular, weak, strong, tolerance and control solutions as its special cases. We state full characterizations of AE solutions for the two-sided interval max-plus systems, including both linear inequalities and linear equations. Moreover, we provide a specific example to illustrate an efficient method of finding the AE solution set.

## Introduction

The max-plus algebra $(\mathbb {R}_{\mathrm{max}}, \oplus, \otimes)$ has appeared under the name extremal algebra for many years [1, 2]. In this algebra, several types of solvability of interval linear systems, including both interval linear equations and interval linear inequalities, have been studied in the literature, see, e.g., weak and strong solvability in [3, 4], tolerance solvability in [5–7], and control solvability in [8]. A detailed discussion of interval solutions can be found in Chap. 6 of [9] and a brief review of the max-plus linear systems in [10].

Two-sided systems of max-plus linear systems containing a vector $x\in \mathbb {R}_{\mathrm{max}}^{n}$ on both sides had been studied by many authors. The theories of these systems in the classical algebra can be found in, e.g., [11–14] and the references therein.

In classical linear algebra, interval linear systems are often used for modeling information systems and engineering problems. Over the past decades, these uncertain systems have been discussed widely, see, e.g., [9, 15–18]. One of the main difficulties when dealing with interval uncertainty is how to understand the concepts of solutions. Shary first proposed the concept of *AE* solutions for interval linear equations in 2002, a rather general concept which includes many traditional concepts of solutions to interval systems as its special cases [19]. Sharaya considered the interval linear system with both equations and inequalities [20]. Recently, Hladík [21] extended the AE solutions to the general interval linear systems of equations, inequalities, or both. Some new results on *AE* solutions can be found in, e.g., [22–24]. Similar methods have been utilized for interval inequalities and interval linear programming for some special cases [25–30].

In this paper, we first extend the concept of AE solutions to max-plus algebra in two-sided interval systems. A practical motivation or studying AE solutions of two-sided interval systems in max-plus algebra is presented in Example 1. Necessary and sufficient conditions for checking the AE solvability of two-sided interval systems in max-plus algebra are formulated in Sects. 4 and 5. Then, in Sect. 6, we present an efficient method to find the AE solution set of two-sided interval linear systems. In Sect. 7, we show that each particular result of weak, strong, tolerance and control solutions established for interval linear systems in existing literature is a special case of the main results of this paper. The conclusion and future application are drawn in Sect. 8.

## Preliminaries

In this section, let us introduce some notations first (see [9]). By a max-plus algebra we understand a triple $(\mathbb {R}_{\mathrm{max}}, \oplus, \otimes)$, where $\mathbb {R}_{\mathrm{max}}=\mathbb {R}\cup\{ \varepsilon\}$, $\mathbb {R}$ is the set of all real numbers and ⊕,⊗ are binary operations defined as

$$a\oplus b=\max\{a, b\},\qquad a\otimes b=a+b, $$

where $\varepsilon=-\infty$. Let ${\mathscr{E}}$ be a matrix consisting entirely of *ε*. The operations ⊕,⊗ are extended to matrices and vectors in the same way as in conventional linear algebra. If $A\in\mathbb {R}_{\mathrm{max}}^{m\times n}, B\in\mathbb {R}_{\mathrm {max}}^{n\times p}$, we define the product $A\otimes B\in\mathbb {R}_{\mathrm{max}}^{m\times p}$ with entries $(A\otimes B)_{ij}$ defined for $i=1,\dots,m, j=1,\dots,p$ as follows:

$$(A\otimes B)_{ij}={\bigoplus_{k= 1} ^{n}}a_{ik}\otimes b_{kj} = \max _{1\leq k\leq n} \{a_{ik} + b_{kj}\}. $$

If $A\in\mathbb {R}_{\mathrm{max}}^{m\times n}, B\in\mathbb {R}_{\mathrm {max}}^{m\times n}$, we define the sum $A\oplus B\in\mathbb {R}_{\mathrm {max}}^{m\times n}$ with entries $(A\oplus B)_{ij}$ defined for $i=1,\dots,m, j=1,\dots,n$ as follows:

$$(A\oplus B)_{ij}= \max\{a_{ij}, b_{ij}\}. $$

An interval matrix is defined as

$$ {\boldsymbol {A}}=[\underline{A},\overline{A}]= \bigl\{ A\in\mathbb{R}_{\mathrm {max}}^{m\times n}| \underline{A}\leq A \leq\overline{A} \bigr\} , $$

where $\underline{A}, \overline{A}\in{\mathbb{R}_{\mathrm{max}}^{m\times n}}$, $\underline{A}\leq\overline{A}$, and “≤” is understood componentwise. An interval vector ${\boldsymbol {b}}=[\underline{b},\overline{b}]=\{b\in{\mathbb {R}_{\mathrm{max}}^{m}}|\underline{b}\leq b \leq\overline{b}\}$ is understood as one-column interval matrix. In the max-plus algebra, the set of all $m\times n$ interval matrices will be denoted by $\mathbb {IR}_{\mathrm{max}}^{m\times n}$, and the set of all *m*-dimensional interval vectors by $\mathbb{IR}_{\mathrm{max}}^{m}$.

Given ${\boldsymbol {A}}\in\mathbb{IR}_{\mathrm{max}}^{m\times n}$ and ${\boldsymbol {B}}\in \mathbb{IR}_{\mathrm{max}}^{m\times n}$, the corresponding two-sided interval linear system of inequalities

2.1 $$ {\boldsymbol {A}}\otimes x \leq{\boldsymbol {B}}\otimes x $$

is the family of systems

2.2 $$ A\otimes x \leq B\otimes x, $$

where $A\in\boldsymbol {A}, B\in\boldsymbol {B}$.

Similarly, the corresponding interval system of equations of the form

2.3 $$ {\boldsymbol {A}}\otimes x = {\boldsymbol {B}}\otimes x $$

represents the set of all systems of linear max-plus systems of the form

2.4 $$ A\otimes x = B\otimes x, $$

where $A\in\boldsymbol {A}, B\in\boldsymbol {B}$.

The following lemmas will be used in the proofs of our results.

### Lemma 2.1

*Let* $A =(a_{ij})\in\mathbb {R}_{\max}^{m\times n}$, $B=(b_{ij}) \in \mathbb {R}_{\max}^{m\times n}$. *If* $A \leq B$, *then* $A\otimes x \leq B\otimes x$.

### Proof

Note that $a_{ij}\leq b_{ij}$, and for $i=1, \dots, m$, we have

$$[A\otimes x]_{i} ={\bigoplus_{j= 1} ^{n}}(a_{ij} \otimes x_{j})={\bigoplus _{j= 1} ^{n}}({a_{ij}+x_{j}})=\max _{1\leq j \leq n}\{a_{ij}+x_{j}\} $$

and

$$[B\otimes x]_{i}={\bigoplus_{j= 1} ^{n}}(b_{ij} \otimes x_{j})={\bigoplus _{j= 1} ^{n}}({b_{ij}+x_{j}})=\max _{1\leq j \leq n}\{ b_{ij}+x_{j}\}. $$

Thus, $[A\otimes x]_{i}\leq[B\otimes x]_{i}$, for $i=1,\dots,m$, i.e., $A\otimes x \leq B\otimes x$. □

### Lemma 2.2

*Let* $A =(a_{ij})\in\mathbb {R}_{\max}^{m\times n}$, $B=(b_{ij}) \in \mathbb {R}_{\max}^{m\times n}$, $C=(c_{ij}) \in\mathbb {R}_{\max }^{m\times n}$. *If* $A \leq C$, *then* $A\oplus B \leq B\oplus C$.

### Proof

Note that $a_{ij}\leq c_{ij}$, we have

$$(A\oplus B)_{ij}= \max\{a_{ij},b_{ij}\} \leq \max \{c_{ij},b_{ij}\} =(B\oplus C)_{ij} $$

for $i=1, \dots, m, j=1, \dots, n$, i.e., $A\oplus B \leq B\oplus C$. □

## AE solutions to interval max-plus systems

Let us recall the concept of *AE* solutions of interval inequalities in classical algebra [19, 21, 22]. The interval matrix can be split as ${\boldsymbol {A}}={\boldsymbol {A}}^{\forall}+{\boldsymbol {A}}^{\exists}$, where ${\boldsymbol {A}}^{\forall}$ is the interval matrix comprising universally quantified coefficients, and ${\boldsymbol {A}}^{\exists}$ concerns existentially quantified coefficients. A vector $x\in\mathbb {R}^{n}$ is an *AE* solution of ${\boldsymbol {A}}x \leq{\boldsymbol {b}}$ if

$$ \forall A^{\forall}\in{\boldsymbol {A}}^{\forall},\ \forall b^{\forall}\in{\boldsymbol {b}}^{\forall},\ \exists A^{\exists}\in{\boldsymbol {A}}^{\exists},\ \exists b^{\exists}\in{\boldsymbol {b}}^{\exists} $$

such that

$$ \bigl(A^{\forall}+A^{\exists} \bigr)x \leq b^{\forall}+b^{\exists}. $$

Analogously, in the max-plus algebra, by using forall-exists quantification of interval parameters, we decompose the interval matrix as ${\boldsymbol {A}} = {\boldsymbol {A}}^{\forall} \oplus{\boldsymbol {A}}^{\exists}$, where the components in the matrix ${\boldsymbol {A}}^{\forall}$ at the positions associated with the existential quantifier are intervals $[\varepsilon, \varepsilon]$ and components in the matrix ${\boldsymbol {A}}^{\exists}$ at the positions associated with the universal quantifier are intervals $[\varepsilon, \varepsilon]$.

Now we extend the concept of AE solutions of an interval linear system in classical algebra to the max-plus algebra.

### Definition 3.1

A vector $x\in\mathbb {R}_{\mathrm{max}}^{n}$ is an AE solution of system ${\boldsymbol {A}}\otimes x\leq{\boldsymbol {B}}\otimes x $(or ${\boldsymbol {A}}\otimes x= {\boldsymbol {B}}\otimes x)$ if for $\forall A^{\forall}\in{\boldsymbol {A}}^{\forall}, \forall B^{\forall}\in {\boldsymbol {B}}^{\forall}, \exists A^{\exists}\in{\boldsymbol {A}}^{\exists}, \exists B^{\exists}\in{\boldsymbol {B}}^{\exists}$ such that

3.1 $$\begin{aligned} &\bigl(A^{\forall}\oplus A^{\exists} \bigr)\otimes x \leq \bigl(B^{\forall}\oplus B^{\exists} \bigr)\otimes x \end{aligned}$$

3.2 $$\begin{aligned} &\bigl(\mbox{or }\bigl(A^{\forall}\oplus A^{\exists} \bigr) \otimes x = \bigl(B^{\forall}\oplus B^{\exists} \bigr)\otimes x \bigr). \end{aligned}$$

The next example demonstrates how this type of solution can be applied to an application problem.

### Example 1

A sportswear company produces *m* types of sport suit, including top and bottom. All the clothes are made from three kinds of material: cotton, polyester fibre and chemical fiber. Due to the varieties of product and material, the production time of each type of suit corresponding to three different kinds of material is different. Each clothing item is finished only after all the material is completed.

Suppose that the production time data of each type of top and bottom, corresponding to three different kinds of material are interval times $[\underline{a_{ij}}, \overline{a_{ij}}]$ and $[\underline{b_{ij}}, \overline{b_{ij}}]$. Before processing, preparing time $x_{j}$ for each material is required. If the working durations $a_{ij}$ and $b_{ij}$ are fixed, the time at which each type of top and bottom are completed is

$$\max \bigl\{ a^{1}_{i1}+x_{1},a^{1}_{i2}+x_{2}, \dots,a^{1}_{in}+x_{n} \bigr\} $$

and

$$\max \bigl\{ b^{1}_{i1}+x_{1},b^{1}_{i2}+x_{2}, \dots,b^{1}_{in}+x_{n} \bigr\} . $$

The packaging should be completed only after the suits, including both tops and the bottoms, are finished.

To optimize production, the company needs to set the preparation time $x_{j}$ for each material such that each type of top and bottom is completed at the same time. This task is equivalent to solving the system of equations

$$\max \bigl\{ a^{1}_{i1}+x_{1},a^{1}_{i2}+x_{2}, \dots,a^{1}_{in}+x_{n} \bigr\} = \max \bigl\{ b^{1}_{i1}+x_{1},b^{1}_{i2}+x_{2}, \dots,b^{1}_{in}+x_{n} \bigr\} , $$

for each $i\in\{1,2,\dots,m\}$, which can be simplified to the matrix form

$$ \begin{bmatrix} a_{11} & a_{12} & \ldots & a_{1n} \\ \vdots & \vdots & & \vdots \\ a_{m1} & a_{m2} & \ldots & a_{mn} \end{bmatrix} \otimes \begin{bmatrix} x_{1} \\ \vdots\\ x_{n} \end{bmatrix} = \begin{bmatrix} b_{11} & b_{12} & \ldots & b_{1n} \\ \vdots & \vdots & & \vdots \\ b_{m1} & b_{m2} & \ldots & b_{mn} \end{bmatrix} \otimes \begin{bmatrix} x_{1} \\ \vdots\\ x_{n} \end{bmatrix} . $$

Under uncertain working durations, it becomes the interval system

3.3 $$\begin{aligned} & \begin{bmatrix} [\underline{a_{11}},\overline{a_{11}}] & \ldots & [\underline {a_{1n}},\overline{a_{1n}}] \\ \vdots & & \vdots \\ [\underline{a_{m1}},\overline{a_{m1}}] & \ldots & [\underline {a_{mn}},\overline{a_{mn}}] \end{bmatrix} \otimes \begin{bmatrix} x_{1} \\ \vdots\\ x_{n} \end{bmatrix} \\ &\quad= \begin{bmatrix} [\underline{b_{11}},\overline{b_{11}}] & \ldots & [\underline {b_{1n}},\overline{b_{1n}}] \\ \vdots & & \vdots \\ [\underline{b_{m1}},\overline{b_{m1}}] & \ldots & [\underline {b_{mn}},\overline{b_{mn}}] \end{bmatrix} \otimes \begin{bmatrix} x_{1} \\ \vdots\\ x_{n} \end{bmatrix} . \end{aligned}$$

In production, the company may not be able to improve parts of its interval production times $[\underline{a_{ij}},\overline{a_{ij}}]$ and $[\underline {b_{ij}},\overline{b_{ij}}]$. In this situation, an AE solution $(x_{1},x_{2},\dots,x_{n})^{T}$ of system (3.3), when $n = 3$, may exist if the company is able to fix the other parts of production times $a_{ij}$ and $b_{ij}$.

## Characterization of AE solutions for two-sided interval linear inequalities

Consider a two-sided interval system of equations of the following form:

$$ {\boldsymbol {A}}\otimes x \leq{\boldsymbol {B}}\otimes x, $$

where ${\boldsymbol {A}}\in\mathbb{IR}_{\mathrm{max}}^{m \times n}$, ${\boldsymbol {B}}\in \mathbb{IR}_{\mathrm{max}}^{m \times n}$.

A sufficient and necessary characterization of AE solutions to interval system of max-plus linear inequalities (2.1) is described in the following theorem.

### Theorem 4.1

*A vector* $x\in\mathbb {R}_{\max}^{n}$ *is an AE solution of two*-*sided interval linear max*-*plus inequalities* (2.1) *if and only if*

4.1 $$ \bigl(\overline{A^{\forall}}\oplus\underline{A^{\exists}} \bigr)\otimes x \leq \bigl(\underline{B^{\forall}}\oplus \overline{B^{\exists}} \bigr)\otimes x. $$

### Proof

Assume that *x* is an AE solution of system ${\boldsymbol {A}}\otimes x \leq{\boldsymbol {B}}\otimes x$. Then for $\forall A^{\forall}\in {\boldsymbol {A}}^{\forall}$, $\forall B^{\forall}\in{\boldsymbol {B}}^{\forall}$, system (2.1) is solvable for some $A^{\exists}=A_{0}^{\exists}$ and $B^{\exists}=B_{0}^{\exists}$. That is, for $\forall A^{\forall}\in{\boldsymbol {A}}^{\forall}$, $\forall B^{\forall}\in{\boldsymbol {B}}^{\forall}$,

$$ \bigl(A^{\forall}\oplus A_{0}^{\exists} \bigr) \otimes x \leq \bigl(B^{\forall}\oplus B_{0}^{\exists} \bigr) \otimes x. $$

Due to the isotone properties presented in Lemmas 2.1 and 2.2, we have

4.2 $$ \bigl(A^{\forall} \oplus\underline{A^{\exists}} \bigr) \otimes x \leq \bigl(A^{\forall }\oplus A_{0}^{\exists} \bigr) \otimes x \leq \bigl(B^{\forall}\oplus B_{0}^{\exists } \bigr) \otimes x \leq \bigl({B^{\forall}}\oplus\overline{B^{\exists}} \bigr) \otimes x. $$

Particularly, letting $A^{\forall}=\overline{A^{\forall}}$ and $B^{\forall}=\underline{B^{\forall}}$ in (4.2), we have

$$ \bigl(\overline{A^{\forall}}\otimes x \bigr)\oplus \bigl( \underline{A^{\exists}}\otimes x \bigr)\leq \bigl(\underline{B^{\forall}} \otimes x \bigr)\oplus \bigl(\overline{B^{\exists }}\otimes x \bigr). $$

Therefore, inequalities (4.1) hold.

Conversely, assume that vector $x\in\mathbb {R}_{\mathrm{max}}^{n}$ satisfies inequalities (4.1). According to Lemma 2.1, for all ${A^{\forall}}\in{\boldsymbol {A}}^{\forall}$, ${B^{\forall}} \in{\boldsymbol {B}}^{\forall}$, we have

$$\bigl(A^{\forall}\oplus\underline{A^{\exists}} \bigr)\otimes x \leq \bigl(\overline {A^{\forall}}\oplus\underline{A^{\exists}} \bigr)\otimes x \leq \bigl(\underline {B^{\forall}}\oplus\overline{B^{\exists}} \bigr) \otimes x \leq \bigl({B^{\forall }}\oplus\overline{B^{\exists}} \bigr) \otimes x. $$

Therefore, for all ${A^{\forall}}\in{\boldsymbol {A}}^{\forall}$, ${B^{\forall}} \in{\boldsymbol {B}}^{\forall}$, there exist $A^{\exists}=\underline{A^{\exists }}$, $B^{\exists}=\overline{B^{\exists}}$ such that the inequalities

$$ \bigl(A^{\forall}\oplus A^{\exists} \bigr)\otimes x \leq \bigl(B^{\forall}\oplus B^{\exists} \bigr)\otimes x $$

hold.

Hence $x\in\mathbb {R}_{\mathrm{max}}^{n}$ is an AE solution of ${\boldsymbol {A}}\otimes x \leq{\boldsymbol {B}}\otimes x$. This completes the proof. □

## Characterization of AE solutions for two-sided interval linear equations

Consider a two-sided interval system of equations of the following form:

$$ {\boldsymbol {A}}\otimes x = {\boldsymbol {B}}\otimes x, $$

where ${\boldsymbol {A}}\in\mathbb{IR}_{\mathrm{max}}^{m \times n}$, ${\boldsymbol {B}}\in \mathbb{IR}_{\mathrm{max}}^{m \times n}$.

A sufficient and necessary characterization of AE solutions to interval system of max-plus linear equations (2.3) is described in the following theorem.

### Theorem 5.1

*A vector* $x\in\mathbb {R}_{\max}^{n}$ *is an AE solution of two*-*sided interval linear max*-*plus equations* (2.3) *if and only if*

5.1 $$\begin{aligned} &\bigl(\overline{A^{\forall}} \oplus\underline{A^{\exists}} \bigr)\otimes x \leq \bigl(\underline{B^{\forall}} \oplus \overline{B^{\exists}} \bigr) \otimes x, \end{aligned}$$

5.2 $$\begin{aligned} & \bigl(\underline{A^{\forall}} \oplus\overline{A^{\exists}} \bigr)\otimes x \geq \bigl(\overline{B^{\forall}} \oplus \underline{B^{\exists}} \bigr) \otimes x. \end{aligned}$$

### Proof

Assume that *x* is an AE solution of system ${\boldsymbol {A}}\otimes x = {\boldsymbol {B}}\otimes x$, then it is an AE solution of ${\boldsymbol {A}}\otimes x \leq{\boldsymbol {B}}\otimes x$ and an AE solution of ${\boldsymbol {B}}\otimes x \leq{\boldsymbol {A}}\otimes x$. Therefore, inequalities (5.1) and (5.2) hold due to Theorem 4.1.

Conversely, assume that vector $x\in\mathbb {R}_{\mathrm{max}}^{n}$ satisfies inequalities (5.1) and (5.2). For the opposite implication, suppose that vector *x* is not an AE solution of ${\boldsymbol {A}}\otimes x={\boldsymbol {B}}\otimes x$. Then, by definition, $\exists\widetilde{A^{\forall}}\in{\boldsymbol {A}}^{\forall}$, $\exists\widetilde{B^{\forall}} \in{\boldsymbol {B}}^{\forall}$, $\forall A^{\exists}\in{\boldsymbol {A}}^{\exists}$, $\forall B^{\exists} \in {\boldsymbol {B}}^{\exists}$ such that

$$ { \bigl(\widetilde{A^{\forall}}\oplus A^{\exists} \bigr) \otimes x \neq \bigl(\widetilde{B^{\forall}}\oplus B^{\exists} \bigr) \otimes x.} $$

Therefore, there exists an $i_{0} \in\{1,\ldots,m\}$ such that

5.3 $$ { \bigl( \bigl(\widetilde{A^{\forall}}\oplus \underline{A^{\exists}} \bigr)\otimes x \bigr)_{i_{0}} > \bigl( \bigl( \widetilde{B^{\forall}}\oplus\overline{B^{\exists }} \bigr)\otimes x \bigr)_{i_{0}}} $$

or

5.4 $$ { \bigl( \bigl(\widetilde{A^{\forall}}\oplus \overline{A^{\exists}} \bigr)\otimes x \bigr)_{i_{0}} < \bigl( \bigl( \widetilde{B^{\forall}}\oplus\underline{B^{\exists }} \bigr)\otimes x \bigr)_{i_{0}}.} $$

If inequality (5.3) is satisfied, due to the isotone property, we have

$$ { \bigl( \bigl(\overline{A^{\forall}}\oplus\underline{A^{\exists}} \bigr)\otimes x \bigr)_{i_{0}} \geq \bigl( \bigl(\widetilde{A^{\forall}} \oplus\underline{A^{\exists }} \bigr)\otimes x \bigr)_{i_{0}} > \bigl( \bigl(\widetilde{B^{\forall}}\oplus\overline {B^{\exists}} \bigr) \otimes x \bigr)_{i_{0}} \geq \bigl( \bigl(\underline{B^{\forall}} \oplus \overline{B^{\exists}} \bigr)\otimes x \bigr)_{i_{0}},} $$

which leads to a contradiction of (5.1).

If inequality (5.4) is satisfied, due to the isotone property, we have

$$ { \bigl( \bigl(\underline{A^{\forall}}\oplus\overline{A^{\exists}} \bigr)\otimes x \bigr)_{i_{0}} \leq \bigl( \bigl(\widetilde{A^{\forall}} \oplus\overline{A^{\exists }} \bigr)\otimes x \bigr)_{i_{0}} < \bigl( \bigl(\widetilde{B^{\forall}}\oplus\underline {B^{\exists}} \bigr) \otimes x \bigr)_{i_{0}} \leq \bigl( \bigl(\overline{B^{\forall}} \oplus \underline{B^{\exists}} \bigr)\otimes x \bigr)_{i_{0}},} $$

which leads to a contradiction of (5.2).

Thus vector *x* is an AE solution of ${\boldsymbol {A}}\otimes x={\boldsymbol {B}}\otimes x$. This completes the proof. □

## Deriving the solution algorithm

From Theorems 4.1 and 5.1, we observe that the equivalent systems of AE solutions are both in a general form of a two-sided systems of max-plus linear inequalities

$$A\otimes x \leq B\otimes x. $$

In this section, we present Example 2 in order to show how to find the AE solution set of a two-sided interval linear system of inequalities ${\boldsymbol {A}\otimes x \leq\boldsymbol {B}\otimes x}$, by using the following theorems proposed in [10]. And this method is also suitable for finding the AE solution set of ${\boldsymbol {A}\otimes x = \boldsymbol {B}\otimes x}$.

### Theorem 6.1

([10])

*Let* $A\in\mathbb{R}_{\max}^{m \times n}$ *and* $B\in\mathbb{R}_{\max }^{m \times n}$. *If there exists an* $i\in M=\{1,2,\dots,m\}$ *such that* $\{j\in N=\{1,2,\dots,n\}:a_{ij}\leq b_{ij}\}=\emptyset$, *then the trivial solution* $x = (\varepsilon, \varepsilon, \dots, \varepsilon)^{T}$ *is a unique solution of system* $A\otimes x = B\otimes x$.

### Theorem 6.2

([10])

*Let* $A=[a_{ij}]$ *and* $B=[b_{ij}]$ *be in* $\mathbb{R}_{\max}^{m \times n}$. *A vector* $x\in\mathbb{R}_{\max}^{n}$ *is a solution of* $A \otimes x \leq B \otimes x$ *if and only if* *x* *belongs to the set*

$$\bigcup_{(j_{i})_{i \in\boldsymbol {M}'}\in H} \bigl\{ x \in\mathbb {R}_{\mathrm {max}}^{n}:C \otimes x \leq x \bigr\} , $$

*where* $H_{i}=\{j \in N:a_{ij} \leq b_{ij}\}$ *for each* $i\in M$, $M'=\{i \in M:H_{i}\neq N\}$, $H=\prod_{i \in\boldsymbol {M}'}H_{i}$, $( j_{i} )_{i\in \boldsymbol {M}'}=( j_{i_{1}},\dots,j_{i_{|\boldsymbol {M}'|}} )$, $i_{1} < \cdots< i_{|\boldsymbol {M}'|}$ *and* $C\in\mathbb{R}_{\max}^{n \times n}$ *has the following components*:

$$ c_{jk}= \textstyle\begin{cases} \varepsilon,&j=k\\ \max_{j_{i}=j,i \in\boldsymbol {M}'}\{a_{ik}-b_{ij}\},& j\neq k. \end{cases} $$

### Theorem 6.3

([10])

*Let* $C=[c_{ij}]\in\mathbb{R}_{\max}^{n \times n}$ *be such that* $c_{ij}=\varepsilon$ *for all* $i=j$. *If* $c_{ij}+c_{ji} > 0$ *for some* $i > j$, *then* $C \otimes x\leq x$ *has no non*-*trivial solution*.

### Theorem 6.4

([10])

*Let* $C=[c_{ij}]\in\mathbb{R}_{\max}^{n \times n}$ *be such that* $c_{ij}=\varepsilon$ *for all* $i=j$ *and* $c_{ij}+c_{ji} \leq0$ *for all* $i > j$. *A vector* $x\in\mathbb{R}_{\max}^{n}$ *is a solution of* $C \otimes x\leq x$ *if and only if* *x* *is a solution of the interval inclusion linear system*

$$Dx\in\boldsymbol {h}, $$

*in which*

$$\begin{aligned} &D= \begin{bmatrix} 1 & -1 & & & & \\ \vdots& & \ddots & & & \\ 1 & & & & & -1 \\ & 1 & -1 & & & \\ & \vdots & & \ddots & & \\ & 1 & & & & -1 \\ & & \ddots & & & \\ & & & 1 & -1 & \\ & & & 1 & & -1\\ & & & & 1 & -1 \end{bmatrix} \in\mathbb{R}^{\frac{n(n-1)}{2}\times n}\quad \textit{and}\\ & \boldsymbol {h}= \begin{bmatrix} [c_{12},-c_{21}] \\ \vdots\\ [c_{1n},-c_{n1}] \\ [c_{23},-c_{32}] \\ \vdots\\ [c_{2n},-c_{n2}] \\ \vdots\\ [c_{n-2,n-1},-c_{n-1,n-2}] \\ [c_{n-2,n},-c_{n,n-2}] \\ [c_{n-1,n},-c_{n,n-1}] \end{bmatrix} \in \mathbb{IR}_{\max}^{\frac{n(n-1)}{2}}. \end{aligned}$$

### Example 2

Consider the system

6.1 $$ \begin{bmatrix} [1,4] & [0,1] & [2,6] \\ [2,4] & [4,6] & [1,3] \\ [3,5] & 3 & [1,4] \\ [2,3] & [0,1] & [2,3] \end{bmatrix} \otimes \begin{bmatrix} x_{1} \\ x_{2} \\ x_{3} \end{bmatrix} \leq \begin{bmatrix} [0,1] & [5,8] & [1,3] \\ [3,5] & [3,4] & [0,1] \\ [0,2] & [0,4] & [1,2] \\ [2,4] & [1,2] & [-2,-1] \end{bmatrix} \otimes \begin{bmatrix} x_{1} \\ x_{2} \\ x_{3} \end{bmatrix}, $$

where

$$\begin{aligned} &A^{\forall}= \begin{bmatrix} [1,4] & [0,1] & \varepsilon\\ \varepsilon & [4,6] & \varepsilon\\ \varepsilon& \varepsilon & \varepsilon\\ [2,3] & [0,1] & [2,3] \end{bmatrix} ,\qquad A^{\exists}= \begin{bmatrix} \varepsilon & \varepsilon& [2,6] \\ [2,4] & \varepsilon & [1,3] \\ [3,5] & 3 & [1,4] \\ \varepsilon & \varepsilon & \varepsilon \end{bmatrix} , \\ &B^{\forall}= \begin{bmatrix} \varepsilon & \varepsilon& [1,3] \\ [3,5] & \varepsilon & [0,1] \\ [0,2] & [0,4] & \varepsilon\\ \varepsilon& \varepsilon & \varepsilon \end{bmatrix} ,\qquad B^{\exists}= \begin{bmatrix} [0,1] & [5,8] & \varepsilon\\ \varepsilon & [3,4] & \varepsilon\\ \varepsilon & \varepsilon & [1,2] \\ [2,4] & [1,2] & [-2,-1] \end{bmatrix} . \end{aligned}$$

By Theorem 4.1, we can obtain the equivalent system of AE solutions,

$$\bigl(\overline{A^{\forall}}\oplus\underline{A^{\exists}} \bigr) \otimes x \leq \bigl(\underline{B^{\forall}}\oplus\overline{B^{\exists}} \bigr) \otimes x, $$

that is,

6.2 $$ \begin{bmatrix} 4 & 1 & 2 \\ 2 & 6 & 1 \\ 3 & 3 & 1 \\ 3 & 1 & 3 \end{bmatrix} \otimes \begin{bmatrix} x_{1} \\ x_{2} \\ x_{3} \end{bmatrix} \leq \begin{bmatrix} 1 & 8 & 1 \\ 3 & 4 & 0 \\ 0 & 0 & 2 \\ 4 & 2 & -1 \end{bmatrix} \otimes \begin{bmatrix} x_{1} \\ x_{2} \\ x_{3} \end{bmatrix}. $$

By Theorem 6.1, we know that the trivial solution $x = (\varepsilon, \varepsilon, \dots, \varepsilon)^{T}$ is not a unique solution of system (6.2), because $\{j\in N=\{1,2,3\}:a_{ij}\leq b_{ij}\}\neq\emptyset$ for each $i\in M=\{1,2,3,4\}$.

Next, by Theorem 6.2, we have $H_{1}=\{2\}, H_{2}=\{1\}, H_{3}=\{3\}, H_{4}=\{1,2\}, M'=\{1,2,3,4\}, H=\prod_{i=1}^{4} H_{i}$ and

$$\bigcup_{(j_{i})_{i \in\boldsymbol {M}'}\in H} \bigl\{ x \in\mathbb {R}_{\mathrm {max}}^{n}:C \otimes x \leq x \bigr\} $$

equals

6.3 $$ \left\{x \in\mathbb {R}_{\mathrm{max}}^{n}: \begin{bmatrix} \varepsilon & 3 & -2 \\ 1 & \varepsilon& 1 \\ 1 & 1 & \varepsilon \end{bmatrix} \otimes x \leq x \right\}\cup \left\{x \in\mathbb {R}_{\mathrm{max}}^{n}: \begin{bmatrix} \varepsilon & 3 & -1 \\ -4 & \varepsilon& -6 \\ 1 & 1 & \varepsilon \end{bmatrix} \otimes x \leq x \right\}. $$

By Theorems 6.3 and 6.4, we derive that the solution set is

6.4 $$ \left\{x \in\mathbb {R}_{\mathrm{max}}^{n}: \begin{bmatrix} \varepsilon & 3 & -2 \\ 1 & \varepsilon& 1 \\ 1 & 1 & \varepsilon \end{bmatrix} \otimes x \leq x \right\}=\emptyset $$

because $c_{21}+c_{12}=4>0$, and the solution set

6.5 $$ \left\{x \in\mathbb {R}_{\mathrm{max}}^{n}: \begin{bmatrix} \varepsilon & 3 & -1 \\ -4 & \varepsilon& -6 \\ 1 & 1 & \varepsilon \end{bmatrix} \otimes x \leq x \right\} $$

equals

$$\begin{aligned} &\bigl\{ x \in\mathbb {R}_{\mathrm{max}}^{n}: Dx\in\boldsymbol {h} \bigr\} , \\ &D= \begin{bmatrix} 1 & -1 & 0 \\ 1 & 0 & -1 \\ 0 & 1 & -1 \end{bmatrix} , \qquad \boldsymbol {h} = \begin{bmatrix} [3, 4] \\ -1 \\ [-6, -1] \end{bmatrix} . \end{aligned}$$

Thus, a vector *x* is an AE solution of system (6.1) if and only if *x* satisfies

6.6 $$ \begin{bmatrix} 3 \\ -1 \\ -6 \end{bmatrix} \leq \begin{bmatrix} 1 & -1 & 0 \\ 1 & 0 & -1 \\ 0 & 1 & -1 \end{bmatrix} x \leq \begin{bmatrix} 4 \\ -1 \\ -1 \end{bmatrix} , $$

for instance, vector $x=(0,-3,1)^{T}$ is an AE solution of system (6.1).

## Special cases of AE solutions of two-sided interval linear systems

As we know, mathematical definitions of various traditional solution types (weak, strong, tolerance, control) of the two-sided interval linear systems of equations $\boldsymbol {A}\otimes x= \boldsymbol {B}\otimes x$ were presented in [10] as follows:

### Definition 7.1

([10])

A vector $x\in \mathbb {R}_{\max}^{n}$ is called

(i) a weak solution of system (2.3) if $A\otimes x = B\otimes x$ for some $A\in\boldsymbol {A}$, $B\in\boldsymbol {B}$;
(ii) a strong solution of system (2.3) if $A\otimes x = B\otimes x$ for all $A\in\boldsymbol {A}$, $B\in\boldsymbol {B}$;
(iii) a tolerance solution of system (2.3) if $A\otimes x = B\otimes x$ for all $A\in\boldsymbol {A}$ for some $B\in\boldsymbol {B}$;
(iv) a control solution of system (2.3) if $A\otimes x = B\otimes x$ for some $A\in\boldsymbol {A}$ for all $B\in\boldsymbol {B}$.

In this section, we first extend the analogous concepts of solutions for two-sided interval linear systems of inequalities $\boldsymbol {A}\otimes x\leq\boldsymbol {B}\otimes x$.

### Definition 7.2

A vector $x\in\mathbb {R}_{\max }^{n}$ is called

(i) a weak solution of system (2.1) if $A\otimes x\leq B\otimes x$ for some $A\in\boldsymbol {A}$, $B\in\boldsymbol {B}$;
(ii) a strong solution of system (2.1) if $A\otimes x\leq B\otimes x$ for all $A\in\boldsymbol {A}$, $B\in\boldsymbol {B}$;
(iii) a tolerance solution of system (2.1) if $A\otimes x\leq B\otimes x$ for all $A\in\boldsymbol {A}$ for some $B\in\boldsymbol {B}$;
(iv) a control solution of system (2.1) if $A\otimes x\leq B\otimes x$ for all $B\in\boldsymbol {B}$ for some $A\in\boldsymbol {A}$.

From the definition of *AE* solutions, it is easy to obtain that the weak, strong, tolerance and control solutions are all special cases of *AE* solutions.

Then we propose the full characterizations of four different types of solution of system $\boldsymbol {A}\otimes x\leq\boldsymbol {B}\otimes x$.

### Corollary 7.1

*A vector* $x \in\mathbb {R}_{\max}^{n}$ *is a weak solution of the interval system* (2.1) *if and only if*

$$ \underline{A}\otimes x \leq\overline{B}\otimes x. $$

### Proof

The assertion follows immediately from Theorem 4.1 if we set ${\boldsymbol {A}}^{\forall}={\mathscr{E}}, {\boldsymbol {B}}^{\forall}={\mathscr{E}}$. □

### Corollary 7.2

*A vector* $x \in\mathbb {R}_{\max}^{n}$ *is a strong solution of the interval system* (2.1) *if and only if*

$$ \overline{A}\otimes x \leq\underline{B}\otimes x. $$

### Proof

The assertion follows immediately from Theorem 4.1 if we set ${\boldsymbol {A}}^{\exists}={\mathscr{E}}, {\boldsymbol {B}}^{\exists}={\mathscr{E}}$. □

### Corollary 7.3

*A vector* $x \in\mathbb {R}_{\max}^{n}$ *is a tolerance solution of the interval system* (2.1) *if and only if*

$$ \overline{A}\otimes x \leq \overline{B}\otimes x. $$

### Proof

The assertion follows immediately from Theorem 4.1 if we set ${\boldsymbol {A}}^{\exists}={\mathscr{E}}, {\boldsymbol {B}}^{\forall}={\mathscr{E}}$. □

### Corollary 7.4

*A vector* $x \in\mathbb {R}_{\max}^{n}$ *is a control solution of the interval system* (2.1) *if and only if*

$$ \underline{A}\otimes x \leq\underline{B}\otimes x. $$

### Proof

The assertion follows immediately from Theorem 4.1 if we set ${\boldsymbol {A}}^{\forall}={\mathscr{E}}, {\boldsymbol {B}}^{\exists}={\mathscr{E}}$. □

Moreover, we find that the equivalent conditions for checking such solvability types of two-sided interval systems of equations in the max-plus algebra formulated in [10] are also special cases in Theorem 5.1.

### Corollary 7.5

([10])

*A vector* $x \in\mathbb {R}_{\max}^{n}$ *is a weak solution of the interval system* (2.3) *if and only if*

$$\begin{aligned} &\underline{A}\otimes x \leq\overline{B}\otimes x, \\ &\overline{A}\otimes x \geq\underline{B}\otimes x. \end{aligned}$$

### Proof

The assertion follows immediately from Theorem 5.1 if we set ${\boldsymbol {A}}^{\forall}={\mathscr{E}}, {\boldsymbol {B}}^{\forall}={\mathscr{E}}$. □

### Corollary 7.6

([10])

*A vector* $x \in\mathbb {R}_{\max}^{n}$ *is a strong solution of the interval system* (2.3) *if and only if*

$$\begin{aligned} &\overline{A}\otimes x \leq\underline{B}\otimes x, \\ &\underline{A}\otimes x \geq\overline{B}\otimes x. \end{aligned}$$

### Proof

The assertion follows immediately from Theorem 5.1 if we set ${\boldsymbol {A}}^{\exists}={\mathscr{E}}, {\boldsymbol {B}}^{\exists}={\mathscr{E}}$. □

### Corollary 7.7

([10])

*A vector* $x \in\mathbb {R}_{\max}^{n}$ *is a tolerance solution of the interval system* (2.3) *if and only if*

$$\begin{aligned} &\overline{A}\otimes x \leq \overline{B}\otimes x, \\ &\underline{A}\otimes x \geq \underline{B}\otimes x. \end{aligned}$$

### Proof

The assertion follows immediately from Theorem 5.1 if we set ${\boldsymbol {A}}^{\exists}={\mathscr{E}}, {\boldsymbol {B}}^{\forall}={\mathscr{E}}$. □

### Corollary 7.8

([10])

*A vector* $x \in\mathbb {R}_{\max}^{n}$ *is a control solution of the interval system* (2.3) *if and only if*

$$\begin{aligned} &\underline{A}\otimes x \leq\underline{B}\otimes x, \\ &\overline{A}\otimes x \geq\overline{B}\otimes x. \end{aligned}$$

### Proof

The assertion follows immediately from Theorem 5.1 if we set ${\boldsymbol {A}}^{\forall}={\mathscr{E}}, {\boldsymbol {B}}^{\exists}={\mathscr{E}}$. □

## Conclusion

We introduced a new concept of AE solutions to two-sided interval linear systems over the max-plus algebra. The full characterizations of AE solutions to the two-sided interval max-plus systems, including both inequalities (2.1) and equations (2.3), were developed. Furthermore, we presented a specific example to illustrate an efficient algorithm of finding the AE solution set of two-sided interval linear systems. The characterizations of several traditional solutions for interval max-plus linear systems are all special cases of of our main results.

An interesting direction for further research is to characterize the so-called EA solutions to two-sided interval linear systems over the max-plus algebra, which can be regarded as dual to AE solutions. In the definition of EA solutions, the separating predicate is such that all the occurrences of the existential quantifier “∃” precede the occurrences of the universal quantifier “∀”. More specifically, a vector $x\in\mathbb {R}_{\mathrm{max}}^{n}$ is an EA solution of system (2.1) (or system (2.3)) if, for $\exists A^{\exists}\in{\boldsymbol {A}}^{\exists}, \exists B^{\exists}\in{\boldsymbol {B}}^{\exists}, \forall A^{\forall}\in{\boldsymbol {A}}^{\forall}, \forall B^{\forall}\in{\boldsymbol {B}}^{\forall}$, (2.2) (or (2.4)) holds. Recently, the characteristics of EA solutions over ordinary interval algebra have just been established [31]. The characterization of EA solutions to interval linear systems over the max-plus algebra remains to be an open problem, which is worth studying further.
